# Increasing the Batch Size of a QESD Crystallization by Using a MSMPR Crystallizer

**DOI:** 10.3390/pharmaceutics14061227

**Published:** 2022-06-09

**Authors:** Jerome Hansen, Peter Kleinebudde

**Affiliations:** Institute of Pharmaceutics and Biopharmaceutics, Heinrich Heine Universitaet Duesseldorf, Universitaetsstrasse 1, 40225 Duesseldorf, Germany; jerome.hansen@hhu.de

**Keywords:** spherical crystallization, continuous crystallization, flowability, solvent fraction, MSMPR crystallizer

## Abstract

Quasi-emulsion solvent diffusion (QESD) crystallizations can improve the micromeritic properties of drugs and excipients. A solution is dispersed in a miscible antisolvent as a transient emulsion. Using this technique, substances that normally crystallize in the form of e.g., needles, agglomerate into spherical, hollow particles. A disadvantage of QESD crystallizations is that the particle size of the agglomerates decreases with an increased solvent fraction of the mother liquor. Therefore, in batch production, many consecutive runs have to be performed, which is a time- and material-intensive process. The aim of this study was to convert a previously used lab-scale batch crystallizer into a mixed-suspension, mixed-product removal (MSMPR) crystallizer, since the batch size could be simply increased by increasing the run time of the system. The mean residence time (MRT) and solvent fraction in the system was predicted and verified using actual measurement curves. The experiments showed that >50 g QESD metformin hydrochloride could be crystallized in a single run, without observing a large shift in the particle size, while maintaining good flowability. Observations regarding the effect of the MRT on the particle size distribution could be verified for the production on a larger scale than previously described.

## 1. Introduction

Transferring a production process from a batch to a continuous mode of operation can yield many benefits; however, certain issues have to be overcome. This transfer is often expensive, and new, multidisciplinary knowledge has to be acquired to understand the complex phenomena happening in a continuous manufacturing line. The continuous production method, therefore, has to provide clear benefits to justify these costs [1]. Processes in batch production are well-defined and experience using these methods is abundantly present. While a large step towards continuous manufacturing has already been achieved, in e.g., the chemical industry, many processes in the pharmaceutical industry still rely on batch production, due to regulatory constraints and reservations [2]. However, the argument can be made that continuous manufacturing is especially suited for the pharmaceutical industry, due to the relatively small batch sizes and high-quality requirements.

Crystallizations are often part of the final purification step in the production of an active pharmaceutical ingredient (API). There are many crystallization methods available, e.g., cooling or antisolvent, which offer different benefits, depending on the product and the required specifications [3]. Quasi-emulsion solvent diffusion (QESD) crystallizations are a type of spherical crystallization that are based on antisolvent crystallizations. A solution is dispersed in a miscible antisolvent, and a transient emulsion is formed [4]. Surfactants are often required to stabilize the emulsion droplets, and to reduce the rate of counter diffusion of the outer and inner phase. A crust is formed at the interface, due to the high-supersaturation present there, upon which further solute crystallizes [5]. QESD crystallizations can be used to create hollow, spherical agglomerates of an API or excipient during crystallization, thereby improving the micromeritic behavior [6]. An advantage of this crystallization method is that the material can be directly compressed into tablets without the need of a further intermediary production step, such as granulation. Disadvantages of QESD crystallizations include the need for large amounts of antisolvent and the use of surfactants during crystallization which can lead to reduced tablet strength [7].

In a previous publication [8], a QESD crystallization method was developed for metformin hydrochloride (MF). This API has very poor flowability and tends to agglomerate when stored [9]. Using a QESD crystallization, metformin hydrochloride is crystallized with an improved flowability and tabletability, while reducing the tendency towards storage agglomeration. The developed production method, however, shows one of the typical limitations of batch antisolvent crystallizations: with increasing batch size, the solvent fraction within the mother liquor increases. This leads to a lower yield and, in the case of QESD crystallizations, to the formation of smaller agglomerates [10]. The increased solvent fraction leads to a reduced diffusion rate of the solvents, which delays the crust formation to a smaller particle size. Therefore, multiple consecutive batches have to be produced to obtain sufficient material for e.g., tableting studies. This is a time- and material-intensive process, due to the intermittent cleaning and set-up steps. The implementation of a continuous crystallizer would, therefore, be advantageous, as batch size can simply be increased by increasing the run time of the crystallizer, without the need for larger equipment, as is often required in batch production. Therefore, the aim of this work was to convert an existing lab-scale batch crystallizer into a simple mixed-suspension, mixed-product-removal (MSMPR) crystallizer, in order to increase the production size of QESD MF, while maintaining the required product specifications.

Antisolvent MSMPR crystallizers are simple continuous crystallizers, where a solution and fresh antisolvent are continuously added to the stirred tank while product slurry is removed. Removal of product slurry can either occur continuously or intermittently, depending on the product attributes and crystallizer setup [1]. It is shown that using a MSMPR crystallizer increases yield [11] and that the recycling of antisolvent is also possible [12].

Tahara et al. [13] demonstrate that QESD crystallizations can be performed using a MSMPR crystallizer, and that product attributes can be controlled by changing system parameters. However, while low flow rates of the API solution are described (~0.01 mL/min), an advantage of the system regarding the suitability to increase the production capacity in a lab scale is not shown. The aim of this study was to produce >50 g QESD MF, with similar product attributes regarding particle size and flowability, compared to the 13 g produced using the batch setup [8]. Furthermore, the authors wanted to give insights into the development of a MSMPR crystallizer for QESD crystallizations, as issues arose that are specific to this type of crystallization.

## 2. Materials and Methods

### 2.1. Materials

Metformin hydrochloride (MF, Auro Laboratories Ltd., Mumbai, India) was dissolved in demineralized water and crystallized in technical-grade acetone (density used for calculations: 0.784 g/mL at 25 °C [14]). Two types of hypromellose (HPMC, Pharmacoat^®^ 603, Shin-Etsu Chemical Co., Ltd., Tokyo, Japan, and Methocel™ K4M, Colorcon, Dartford, UK) were used as polymeric stabilizers of the quasi-emulsion.

For tableting, hyprolose (HPC SSL SFP, Nippon Soda, Tokyo, Japan) was used as a dry binder, and magnesium stearate (Parteck^®^ LUB MST, Merck KGaA, Darmstadt, Germany) as a lubricant.

Caffeine (BASF, Ludwigshafen, Germany) was used as a tracer, to determine the residence time distribution.

### 2.2. Mixed-Suspension, Mixed-Product Removal (MSMPR) Crystallizer

**Figure 1:**
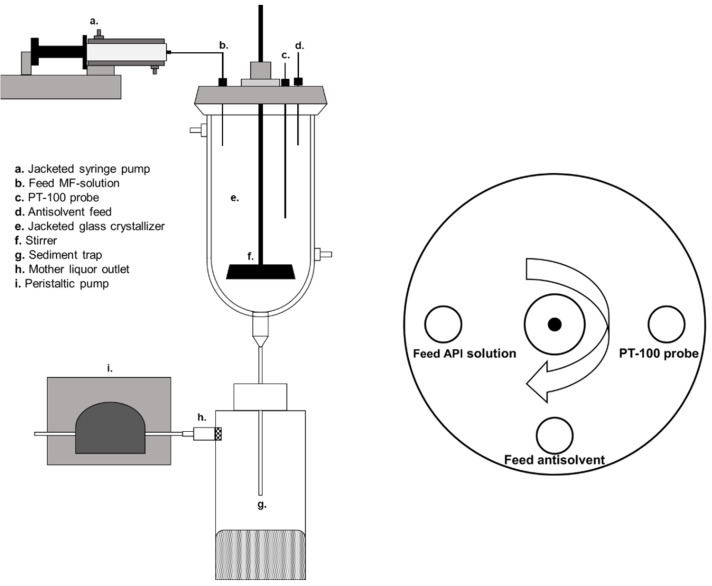
(**left**): MSMPR setup used for QESD crystallizations (the peristaltic pump (i.) is also used to pump fresh antisolvent into the crystallizer through the antisolvent feed line (d.)), (**right**): top view of the ports in the lid of the crystallizer with the direction of stirring (clockwise).

**Figure 2:**
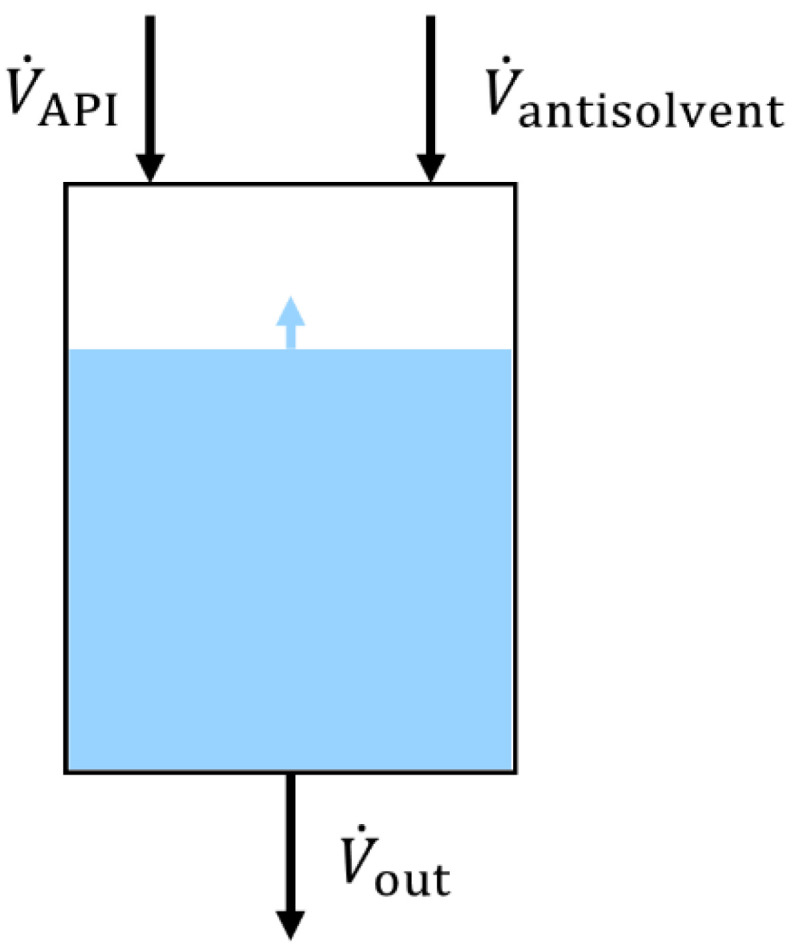
Schematic representation of the MSMPR crystallizer.

The MSMPR crystallizer was set up using the 1 L jacketed, round-bottomed glass crystallizer (ID = 80 mm) equipped with an overhead stirrer (Eurostar 60 control, IKA, Staufen im Breisgau, Germany) and used in the previous publications, as seen in Figure 1 [7,8]. The crystallizer was thermally controlled using a circulating thermostat (DD-200F, Julabo, Seelbach, Germany) that controlled the antisolvent temperature to 20 °C. The fresh antisolvent was kept at room temperature (~23 °C) before being pumped into the system. Stirrers with a diameter of 50 mm were used. A PTFE-coated, four-bladed, pitched (45°) impeller was used, if not stated otherwise. The stirrers were positioned 2 cm from the bottom of the crystallizer. The just-suspended-state of the particles was determined visually, and a rotational speed of >185 rpm was required to keep all particles in motion [8]. MF solutions were fed using a jacketed syringe pump (Legato 100, KD Scientific, Holliston, MA, USA) set to 55 °C, equipped with 0.75 mm ID ETFE tubing (20 cm length). If volumes >50 mL were pumped, the syringe had to be interchanged with a freshly filled syringe; this took 1–2 min on average. For all experiments, the agglomerates were collected in a sediment trap located at the outlet at the bottom of the crystallizer. The sediment trap consisted of a 650 mL polyethylene bottle with a 4 mm ID downpipe. The agglomerates sedimented to the bottom of the trap, while the mother liquor above the outlet of the down pipe remained clear. The mother liquor outlet consisted of a stainless-steel flange, in which a stainless-steel filter (pore size = 40 µm) was integrated to avoid the removal of agglomerates from the trap. The simultaneous removal of the mother liquor (h. in Figure 1), and the addition of fresh antisolvent (d. in Figure 1), occurred using a single peristaltic pump (IPC-8, Ismatec SA, Opfikon, Switzerland) equipped with Tygon^®^ pump tubing (MHLL 2765-175, 2.79 mm ID), as no other pumps with similar flow rates were available. This results in identical flow rates for ${\dot{V}}_{\mathrm{out}}$ and ${\dot{V}}_{\mathrm{antisolvent}}$, shown in Figure 2, so that true steady-state operation was not be achieved. Polyethylene tubing (3 mm ID, 4 mm OD) was used as transfer lines for the antisolvent and mother liquor, as it has a high chemical resistance towards acetone, and is low in cost. Filtration of the agglomerates and washing with fresh antisolvent (acetone) occurred at the end of each trial on stainless-steel sieves (63 µm mesh size), after which the agglomerates were dried overnight at 60 °C.

### 2.3. MSMPR QESD Crystallization of Metformin Hydrochloride

The MF solution was prepared by dissolving HPMC (1.41% (*w*/*w*) Pharmacoat^®^ 603, unless stated otherwise) in water, followed by the addition of MF (42.3% (*w*/*w*)), and heating to 54 °C. This corresponded to a volumetric solvent fraction of 61.4% (*v*/*v*) for the MF solution. Volumes of 35.5 mL (=13 g QESD MF), 50 mL (=19 g QESD MF), 100 mL (=38 g QESD MF), and 150 mL (=55 g QESD MF) were crystallized. Before a crystallization trial, the crystallizer and sediment trap were filled with acetone. Fill levels of 300, 450, and 600 g were used in the crystallizer. The peristaltic pump was calibrated (*n* = 3) for flow rates of ${\dot{V}}_{\mathrm{antisolvent}}$ and ${\dot{V}}_{\mathrm{out}}$ (Figure 2) at either 23.80, 17.92, or 12.04 mL/min. The crystallizer was allowed to equilibrate for 15 min before the start of each crystallization trial. The MF solution was pumped into the crystallizer at a rate of 2.5 mL/min (${\dot{V}}_{\mathrm{API}}$, Figure 2), which corresponds to a ${\dot{V}}_{\mathrm{water}}$ of 1.53 mL/min. As ${\dot{V}}_{\mathrm{out}}$ = ${\dot{V}}_{\mathrm{antisolvent}}$, the fill level of the crystallizer increased at the rate of ${\dot{V}}_{\mathrm{API}}$. The transfer line between the crystallizer and the sediment trap was constantly monitored, to ensure that no blockage of the line occurred. After the addition of the desired volume of MF solution occurred, the crystallizer was run for one further mean residence time, before increasing the flow rate of ${\dot{V}}_{\mathrm{out}}$ to the maximum rate of the pump to collect all material in the sediment trap. The reproducibility of the system was tested by performing a crystallization at set parameters in triplicate; all other crystallizations were performed once.

### 2.4. Determination of Residence Time Distribution (RTD) and Calculation of Solvent Fraction

**Figure 3:**
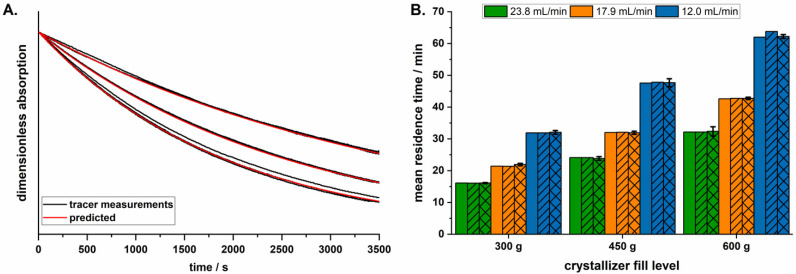
(**A**). Example of actual single tracer measurements curves (*n* = 3 at each pump rate) at a fill level of 600 g with the predicted curves; (**B**). predicted MRTs at t = 0 min, using different equations (without pattern: Equation (2), dash: Equation (3)) compared to measured MRTs (crossed pattern, mean ± s, *n* = 3).

The RTD of particles in MSMPR crystallizers is commonly determined by running only demineralized water through the system at the set parameters used during crystallization. A pulse tracer is added, and its concentration determined continuously within the system. Onyemelukwe et al. [15] described the use of sodium chloride as a tracer and determining the RTD using conductivity measurements. For this publication, 1 mL caffeine solution (1%, *w*/*w*) was added using an Eppendorf pipette as a UV–Vis tracer and the absorption was measured continuously (λ = 273.0 nm, 5 mm flow through cuvette, UV-1800, Shimadzu, Kyoto, Japan) for 3600 s at 0.2 Hz. The measurements revealed a first-order kinetic (Figure 3A), so that the elimination constant (*k_e_*) for a respective flow rate of the peristaltic pump (${\dot{V}}_{\mathrm{out}}$) and fill level of the crystallizer (*V*) could be calculated using Equation (1).

(1) $$k_{e}=\frac{{\dot{V}}_{\mathrm{out}}}{V}$$

The accurate prediction of the *k_e_* at *V*_0_ was verified by comparing the actual single measurement curves of the tracer at a constant fill level to the predicted curves (Figure 3A).

To verify the accurate prediction of the MRT, two equations were evaluated and compared to the MRT obtained from the tracer measurements. Predictions of the MRT were performed using both the predicted *k_e_* values (Equation (2)) and Equation (3), which describes the MRT in ideal continuous stirred-tank reactors [16]. These were compared to the values obtained using the actual tracer measurements (Figure 3B). As there were only small differences between the predicted and measured MRT values, Equation (3) was used for further calculations, due to its simplicity.

(2) $$\mathrm{MRT}=\frac{\int_{0}^{\infty }t*e^{-k_{e}*t}\;dt}{\int_{0}^{\infty }e^{-k_{e}*t}\;dt}$$

(3) $$\mathrm{MRT}=\frac{V}{{\dot{V}}_{\mathrm{out}}}$$

Since the inward flow of fresh acetone (${\dot{V}}_{\mathrm{antisolvent}}$) and the outward flow of the mother liquor (${\dot{V}}_{\mathrm{out}}$) were equal, as they were controlled by the same peristaltic pump, the volume of mother liquor increased by the flow rate of the MF solution (${\dot{V}}_{\mathrm{API}}$) over time. The fill level of the crystallizer, at any time point, can be calculated using Equation (4), using the initial fill level (*V*_0_).

(4) $$V_{t}=V_{0}+{\dot{V}}_{\mathrm{API}}*t$$

Therefore, the elimination constant ($k_{e}{}_{t}$, Equation (5)) and the MRT change during the crystallization, and have to be calculated for each time point, as they depend on the current fill level of the crystallizer. Calculating the average MRT of the agglomerates over the course of the crystallization was done using Equation (6).

(5) $$k_{e}{}_{t}=\frac{{\dot{V}}_{\mathrm{out}}}{V_{t}}$$

(6) $$MRT_{\mathrm{average}}=\frac{\int_{0}^{t}\frac{V_{t}}{{\dot{V}}_{\mathrm{out}}}dt}{t}$$

The solvent fraction (SF) of the mother liquor, i.e., the fraction of water, at any time point during the batch crystallization can be calculated using the volumetric flow rate of the water fraction (${\dot{V}}_{\mathrm{water}}$, Equation (7)) from the aqueous solution, using Equation (8).

(7) $${\dot{V}}_{\mathrm{water}}={\dot{V}}_{\mathrm{API}}*\varphi_{\mathrm{water}}$$

(8) $$SF_{\mathrm{batch},t}\;\left(V/V\right)=\frac{{\dot{V}}_{\mathrm{water}}}{V_{0}+{\dot{V}}_{\mathrm{API}}*t}$$

The SF of the mother liquor during crystallization using the MSMPR setup could be calculated using Equation (9), further simplified to Equation (10) by using Equation (5) to calculate the elimination constant at every time point.

(9) $$SF_{t}\;\left(V/V\right)=\frac{{\dot{V}}_{\mathrm{water}}}{k_{e}{}_{t}*V_{t}}*\left(1-e^{-k_{e}{}_{t}*t}\right)$$

(10) $$SF_{t}\;\left(V/V\right)=\frac{{\dot{V}}_{\mathrm{water}}}{{\dot{V}}_{\mathrm{out}}}*\left(1-e^{-k_{e}{}_{t}*t}\right)$$

### 2.5. Particle Size Distribution (PSD)

Dynamic image analysis was used to measure the PSD of the QESD MF agglomerates (Haver CPA 2-1, Haver & Boecker OHG, Oelde, Germany). The feeder was equipped with an ultrasonic dispersion unit. For each measurement, the PSD of the entire batch was determined (*n* = 1).

### 2.6. Microscopy

A light microscope (Leica DMLB, Leica Camera AG, Wetzlar, Germany) equipped with a camera was used to obtain microscopic images of the agglomerates.

### 2.7. Flowability

The Hausner ratio (HR) of the MF agglomerates was calculated by determining the bulk and tapped densities (after 1250 taps) using the apparatus described by the European Pharmacopoeia 10.0 (2.9.34.; *n* = 3) [17], using a 250 mL graduated cylinder. The HR was calculated using the bulk volume (BV) and tapped volume (TV) (HR = BV/TV).

The angle of repose was determined according to the European Pharmacopoeia 10.0 (2.9.36.; *n* = 3) [18], using an 8 cm diameter base plate, with 10 g MF agglomerates.

### 2.8. Earth Mover’s Distance (EMD) for the Comparison of PSDs

The earth mover’s distance, also known as the Wasserstein distance, described by Hu et al. [19], was used to compare the PSDs of different QESD MF agglomerate batches. The EMD values were calculated using an in-house-developed Python script. It describes the similarity of a reference distribution profile to a test distribution profile by calculating the mean distance between the curves. The variability of the system is measured by averaging the distance between single reference curves and the mean reference curve, in order to obtain a reference value. The EMD between different distribution profiles can then be calculated and compared to the reference value. As EDM values are only comparative, no limits are defined for a definition of similarity or dissimilarity. A comparison with the reference value is therefore essential. An increase in the EDM value shows that two curves are more dissimilar.

### 2.9. Tableting

Blending the QESD MF agglomerates and the reference material with 5% (*w*/*w*) HPC was performed in a Turbula mixer type T2C (Willy Bachofen, Muttenz, Switzerland) for 20 min, and then for further 2 min after 0.5% (*w*/*w*) magnesium stearate was added as a lubricant. The reference MF had to be freshly milled using a 1 mm friction sieve (BTS 100, L.B. Bohle GmBH, Ennigerloh, Germany), due to the agglomeration of the material.

The tablets were produced using a compaction simulator (Styl’One Evolution, Medelpharm, Beynost, France) equipped with 11.28 mm flat-faced punches. The die was filled manually. Tablets were produced at a range of 100–250 MPa, and weighed approximately 260–300 mg. The tablet press was operated using an asymmetrical main compression in force mode. The strength of the tablets was determined after 24 h storage under climate conditions (45% RH, 21 °C). The weight, dimensions, and required breaking force were measured using a tablet tester (Smart Test 50, Sotax AG, Basel, Switzerland, *n* = 6), and the data used to calculate the tensile strength [20] of the tablets.

## 3. Results and Discussion

### 3.1. Residence Time Distribution (RTD) and Solvent Fraction (SF)

As the fill level of the developed MSMPR crystallizer increased during a crystallization trial, the RTD was only determined using tracer measurements for the time point t = 0 min, and these measurements were used to check the predicted values. The measurements of the RTD using the single pulse method showed a first-order elimination kinetic from the crystallizer (Figure 3A). The RTD was determined at three different fill levels (300, 450, and 600 g acetone), at three different pump rates of the peristaltic pump (12.0, 17.9, and 23.8 mL/min). Overlaying the predicted curves over the actual single measurement curves (Figure 3A) showed that the elimination constant (*k_e_*) and, therefore, the MRT, could be accurately predicted. The MRT could be predicted using both Equations (2) and (3), and showed little deviation from the values calculated using the tracer experiments (Figure 3B). Due to its simplicity, Equation (3) was therefore used for further calculations. The average MRT of the particles over the course of a crystallization trail was calculated using Equation (6). The increase in fill level of the crystallizer caused an increase in the MRT of the agglomerates. For example, the MRT_average_ during the longest crystallization trial (using 150 mL MF solution) was calculated to be 35.3 min, while the initial MRT was measured at 32.4 ± 1.4 min.

**Figure 4:**
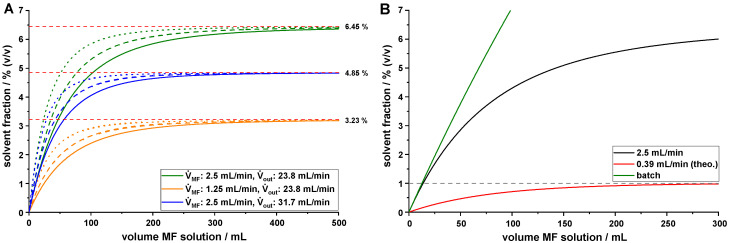
(**A**). Examples of calculated solvent fraction during QESD MF crystallization with different *V*_0_ (straight lines: 600 mL, dashed lines: 450 mL, dotted lines: 300 mL); (**B**). comparison of solvent fraction between batch and MSMPR setup at *V*_0_ = 600 g acetone and ${\dot{V}}_{\mathrm{out}}$ = 23.8 mL/min for the MSMPR trials.

Due to the constant exchange of mother liquor with fresh antisolvent, the solvent fraction (SF) during a MSMPR QESD crystallization was lower than during batch operation (Figure 4B). The maximum solvent fraction during MSMPR crystallization was only dependent on the ratio between ${\dot{V}}_{\mathrm{API}}$ and ${\dot{V}}_{\mathrm{out}}$. The initial fill volume of the crystallizer only affects the time point at which steady state is achieved (Equation (1)). An overview of the influence of the process parameters on the SF is given in Figure 4A. Nocent et al. [21] recommended a maximum solvent fraction of 1% for QESD crystallization, as exceeding this threshold led to the formation of sticky agglomerates for albuterol sulfate. This value probably has to be evaluated independently for each system, as it could depend on e.g., the solubility of the API in the mother liquor. 

At a certain fill level, setting the SF should ideally be achieved by adjusting the flow rate of the API solution; however, low flow rates can lead to crystallization of material in the line. An alternative would be to change the elimination pump rate (${\dot{V}}_{\mathrm{out}}$); however, as this directly effects the MRT of agglomerates, other product attributes would change as well [13]. Running the crystallizer at its maximum fill level (600 g acetone) and the highest analyzed ${\dot{V}}_{\mathrm{out}}$ of 23.8 mL/min resulted in a maximum solvent fraction of ~6.5% (*v*/*v*) at a ${\dot{V}}_{\mathrm{API}}$ of 2.5 mL/min. A reduction in the pump rate of the MF solution was not possible, as the material crystallized within the ETFE tubing. Calculations revealed that a theoretical pump rate of 0.39 mL/min ensures a MSMPR crystallization process with a solvent fraction of ≤1% (Figure 4B).

**Figure 5:**
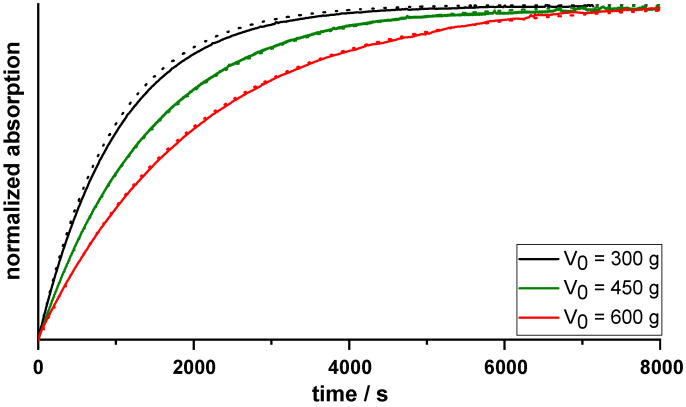
Normalized absorption curves during continuous addition of tracer solution (0.1 mL/min, solid lines) vs. predicted curves (dotted lines), at constant ${\dot{V}}_{\mathrm{out}}$ = 23.80 mL/min, and three different fill levels (n = 1).

To verify whether the equation developed for the determination of the SF was actually suitable, three trials were conducted where the tracer solution (1% caffeine) was continuously pumped into the crystallizer (0.1 mL/min) using the syringe pump and the absorption was continuously measured. A constant ${\dot{V}}_{\mathrm{out}}$ of 23.80 mL/min was set and three different initial fill levels were tested. Differences between the actual measurement and predicted curves were minimal (Figure 5), so the equation was assumed to be valid.

### 3.2. Reproducibility of MSMPR Batches

**Figure 6:**
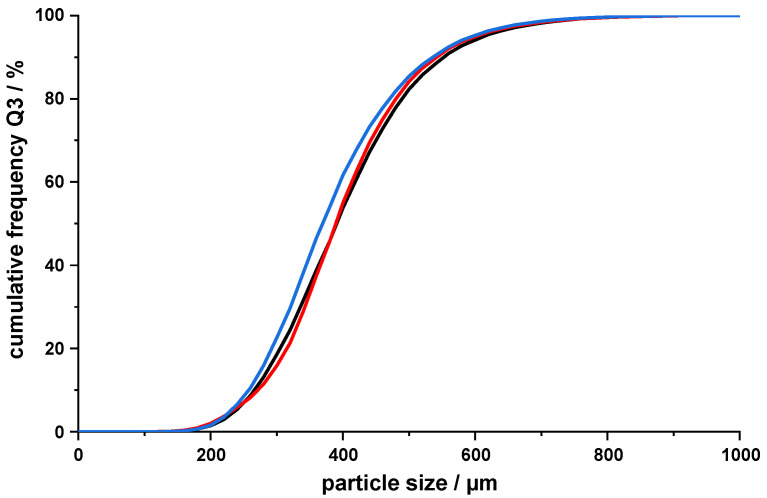
PSD of three MSMPR batches produced under identical conditions (*n* = 1, entire batch).

At first, an evaluation regarding the uniformity of batches produced using the MSMPR crystallizer was performed by regarding the PSDs of three batches produced under identical conditions (*V*_0_ = 450 g, 350 rpm, ${\dot{V}}_{\mathrm{API}}$ = 2.5 mL/min, and ${\dot{V}}_{\mathrm{out}}$ = 17.9 mL/min). Between each batch, the crystallizer was completely disassembled and all components cleaned extensively. As defined previously for the batch process [8], deviations from the mean of <5% at d10, d50, and d90 would be seen as tolerable. These criteria were met by the three produced batches (Figure 6, largest single deviation = 3.7% at d50). The reference EMD value was calculated to be 4.2 ± 1.4 for this system.

### 3.3. Considerations When Developing a MSMPR Crystallizer for QESD Crystallizations

An abundance of publications are available in the literature that describe the use of continuous crystallizers for cooling or antisolvent crystallizations [22,23,24]; however, there are only a few that describe continuous QESD crystallizations [25,26]. Furthermore, to the best of our knowledge, only a single publication exists for QESD crystallizations using a MSMPR setup [13]. Wood et al. [24] released a review detailing challenges and how to overcome them in the set-up of continuous crystallizers; however, there are no specific guidelines available for MSMPR QESD crystallizations. Converting a batch crystallizer into a MSMPR crystallizer for QESD crystallizations revealed several specific issues regarding the set-up and crystallization procedure. The main issues during development could be grouped into transfer line blockage, choice of filtration method and stirrer type/encrustation.

#### 3.3.1. Choosing a Suitable Transfer Pump for the Addition of the MF Solution

Choosing a suitable pump and setup for the transfer of the heated API solution into the crystallizer can be difficult. The cooling of the API/surfactant solutions in the transfer lines can cause precipitation of the material and subsequent blockage of the tubes. Therefore, the lines need to be as short as possible. It is recommended to keep solutions at 20 °C above their saturation point [15]; however, this cannot always be implemented. Especially in the case of QESD crystallization, the maximum temperature of the API solution is limited by the polymeric stabilizer used, if it is present within the solvent phase. Hypromellose, for example, has a cloud point between 50 and 70 °C, above which the polymer is no longer freely soluble, and, therefore, cannot stabilize the transient emulsion [27]. A previously developed viscosity-based screening technique for QESD stabilizers [27] could be used to identify a different HPMC type with a higher cloud point, which can ease the transfer of the solution. One should also keep in mind that the PSD of QESD agglomerates depends on the viscosity of the solution, which decreases with increasing temperature. 

Peristaltic pumps are often used to transfer the API solutions into crystallizers [15,28,29]. However, they require relatively long lines (>50 cm), which often clogged during operation, even when using tubing with an ID of only 1.42 mm. Instead, a syringe pump was used, as its outlet can be positioned directly at the inlet of the crystallizer, thereby reducing the length of the required ETFE tubing (<25 cm). To ensure the constant temperature of the MF solution, a stainless-steel, jacketed 50 mL glass syringe was developed and built by the fine mechanic’s department of the university. The flowrate of the 42.3% MF solution was still limited to ≥2.5 mL/min, as material would otherwise build up at the outlet of the ETFE tubing, which would clog the line. It was also important to freshly cut the end of the tubing before each trial to avoid buildup of the material. Due to the limited size of the glass syringe (50 mL), it had to be filled multiple times during a larger run, so that the system was not truly continuous.

#### 3.3.2. Continuously Removing Product from the Crystallizer

**Figure 7:**
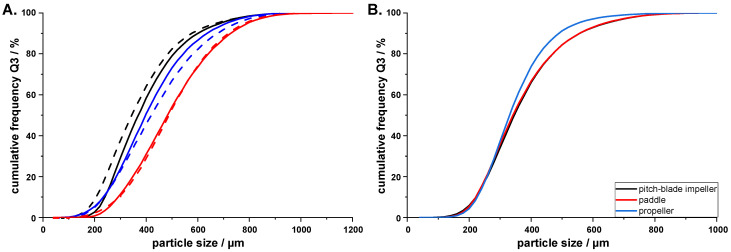
(**A**). PSD of three different QESD MF agglomerate batches before (dotted line) and after (solid line) passing through a peristaltic pump (*n* = 1, entire batch); (**B**). PSD of QESD MF agglomerates produced with different stirrer types under constant conditions (*V*_0_ = 300 g, ${\dot{V}}_{\mathrm{out}}$ = 23.8 mL/min, 300 rpm, n = 1, entire batch).

Continuously removing the suspension from the crystallizers posed another challenge. The combined pressure/vacuum system developed by Hou et al. [30] was not available to the authors, but it has been effective in transferring agglomerates created by the spherical agglomeration method [31]. In literature, peristaltic pumps are commonly used to transfer the slurry from MSMPR crystallizers [12,15,32]; however, since the agglomerates produced during QESD crystallizations are hollow and therefore more fragile than regular crystals, it was unclear whether the rolls of the peristaltic pump would crush the material. A simple trial revealed that a milling effect of a peristaltic pump on three different QESD MF agglomerate batches was not observed when comparing the PSD of QESD MF agglomerates before and after being suspended in acetone and pumped through the peristaltic pump (Figure 7A). The EMD values between the curves are 16.3 (black curve), 9.5 (blue curve), and 1.3 (red curve).

Nevertheless, a setup was chosen where a sediment trap was located before the peristaltic pump. This allowed for the collection of the entire material before filtering and washing it in a single step. Pumping the suspension directly on to the stainless-steel sieve was also possible; however, more difficult in practice, as the particles have to be washed regularly using antisolvent to avoid caking due to residual solute present in the mother liquor, which crystallizes during drying. A possible solution to this has been presented by Liu et al. [33], who developed a filtration carousel equipped with several stations, where the particles can be collected, washed and dried.

#### 3.3.3. Influence of Stirrer Type and Material

**Figure 8:**
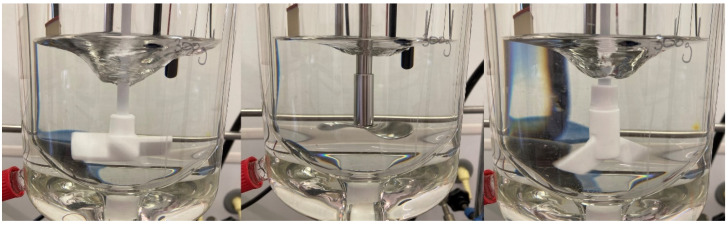
Vortex produced by different stirrer types at 300 rpm.

The influence of the stirrer type on the QESD crystallization of drugs has not yet been discussed in literature. Irrespective of the shape, the use of PTFE-coated stirrers is recommended by the authors, as encrustations were more prominent on the stainless-steel stirrer used. The three analyzed stirrers are seen in Figure 8 (four-bladed, pitch-blade, PTFE-coated impeller (left); three-bladed, stainless-steel propeller (middle); and two-blade PTFE-coated paddle (right)) and all have a diameter of 5 cm, so that a constant tip speed was ensured. No difference in the PSD of the QESD MF agglomerates could be observed when using the paddle and pitch-blade impeller (Figure 7B, EMD = 1.2). However, the agglomerates produced with the propeller were surprisingly smaller, even though the vortex created was much shallower (Figure 8), indicating a reduced radial flow (EDM to pitched-blade impeller = 9.5) and lower shear forces [34,35]. A possible explanation could be that collisions with the harder stainless-steel stirrer caused more fragmentation of the agglomerates than with the softer PTFE-coated blades.

#### 3.3.4. Choice of Polymeric Stabilizer

With the help of a previously developed viscosity-based QESD screening technique [27], Methocel™ K4M (0.13% (*w*/*w*)) was identified as an alternative polymeric stabilizer for the spherical crystallization of MF. The reduction in the amount of polymer required, and the resulting increased drug load of the agglomerates [27], as well as the higher cloud point compared to Pharmacoat^®^ 603, might be an incentive to use K4M as a stabilizer for the MSMPR setup; however, this formulation was more problematic to produce using the MSMPR setup.

**Figure 9:**
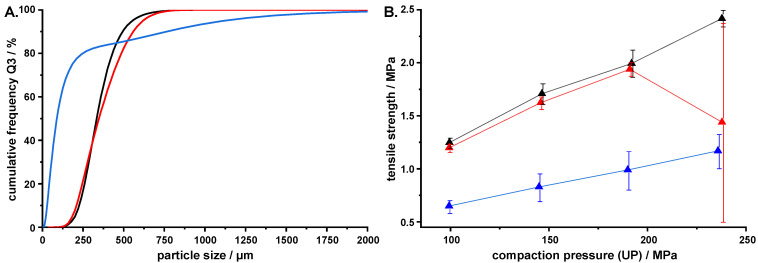
(**A**) PSD (*n* = 1, entire batch); (**B**) tensile strength of tablets produced (*n* = 6, mean ± s) from QESD MF using different hypromellose types as polymeric stabilizers (black: Pharmacoat^®^ 603, red: Methocel™ K4M) compared to the freshly milled reference material (blue).

Just as in the previous study, almost no change in the PSD of the agglomerates was observed when using the MSMPR setup to crystallize the solutions containing K4M or Pharmcoat^®^ 603 as a polymeric stabilizer (*V*_0_ = 600 g, 350 rpm, ${\dot{V}}_{\mathrm{API}}$ = 2.5 mL/min, ${\dot{V}}_{\mathrm{out}}$ = 17.9 mL/min, EDM = 8.5; Figure 9A). During the crystallization, however, more adherence of particles to the walls of the crystallizer and the stirrer was observed when using K4M. Intermittent blockage of the tubing between the crystallizer and the sediment trap was also observed. As K4M has a higher nominal viscosity (4000 mPa·s) than Pharmacoat^®^ 603 (3 mPa·s), the precipitated HPMC particles could be stickier if they remain partially swollen from residual water, which could lead to a stronger adherence to the surfaces in the crystallizer. 

Determining the strength of the produced tablets revealed that both formulations of QESD MF behaved similarly up to a compaction pressure of approximately 200 MPa (Figure 9B). As previously described, the QESD agglomerates showed an improved tabletability compared to the reference material [7]. At >200 MPa, however, capping of the tablets produced with K4M QESD MF was observed, which led to the large standard deviation of this data point in Figure 9B. The reason for this might be the reduced HPMC content of these agglomerates (K4M: 0.37%, Pharmacoat^®^ 603: 2.19% [27]), as HPMC has binder qualities [36]. Therefore, more dry binder would be required during tableting, so that the overall drug load of the tablets would not be considerably higher.

### 3.4. Influence of MRT on Agglomerate Size

**Figure 10:**
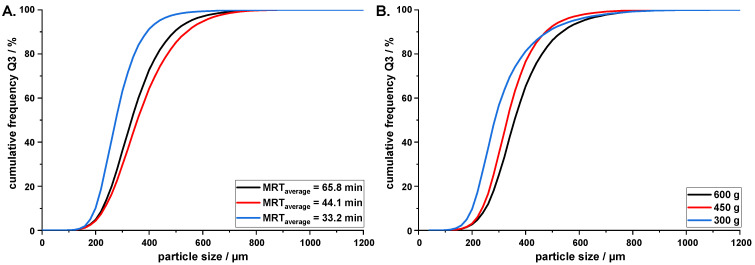
(**A**). PSD of QESD MF agglomerates produced at different MRTs by adjusting ${\dot{V}}_{\mathrm{out}}$ at *V*_0_ = 600 g; (**B**). similar average MRTs of ~33.5 min at otherwise constant conditions (350 rpm, ${\dot{V}}_{\mathrm{API}}$ = 2.5 mL/min, 50 mL MF solution, n = 1, entire batch).

With all other system parameters left constant, the MRT of the agglomerates in the crystallizer can influence the particle size of the agglomerates [13]. With increasing MRT of QESD agglomerates within the crystallizer, crystal growth due to the supersaturation of the mother liquor and agglomeration occurs, which leads to an increase in the particle size. This could be seen when increasing the MRT_average_ from 33.2 to 44.1 min (EMD = 44, Figure 10A). A further increase in the MRT_average_ to 65.8 min led to a slight reduction in the particle size (EMD = 32, compared to MRT_average_ = 33.2 min), as fragmentation likely occurred, due to collusions of the agglomerates with the wall, stirrer, and other particles. A short MRT is favorable, as the production time can therefore be reduced and the solid fraction in the crystallizer decreases.

For scaling purposes, one method of maintaining the particle size during transfer is to maintain the MRT; however, this strategy is unsuitable for MSMPR QESD crystallizations (Figure 10B). Even though the MRT_average_ was kept similar (*V*_0_ 600 g = 33.2 min, *V*_0_ 450 g = 33.5 min, and *V*_0_ 300 g = 34.0 min), a shift to smaller particle sizes was observed when reducing the initial fill level of the crystallizer from 600 to 450 g (EMD = 16). A further reduction was visible when the fill level was further decreased to 300 g (EMD = 37). This could be explained by the increased input of mechanical energy into the system, when the stirrer rpm is kept constant while decreasing the initial fill level of the crystallizer. An increase in the energy input leads to smaller emulsion droplets, which results in smaller agglomerates. Furthermore, the reduction in ${\dot{V}}_{\mathrm{out}}$, to maintain the MRT at lower fill levels, resulted in a higher solvent fraction present in the mother liquor (Equation (10)). While the average SF throughout the entire crystallization at *V*_0_ = 600 g was calculated to be 1.59%, reducing *V*_0_ to 300 g resulted in an average SF of 3.08%, which led to smaller agglomerates. When up-scaling a MSMPR QESD crystallizer, it is, therefore, important to adjust ${\dot{V}}_{\mathrm{API}}$ according to Equation (10) in order to maintain the SF, if possible. Adjusting the stirrer rpm or diameter to maintain the droplet size cannot be calculated easily, as computational fluid dynamics are required to ensure constant shearing within the crystallizer.

### 3.5. Increasing Batch Size

**Figure 11:**
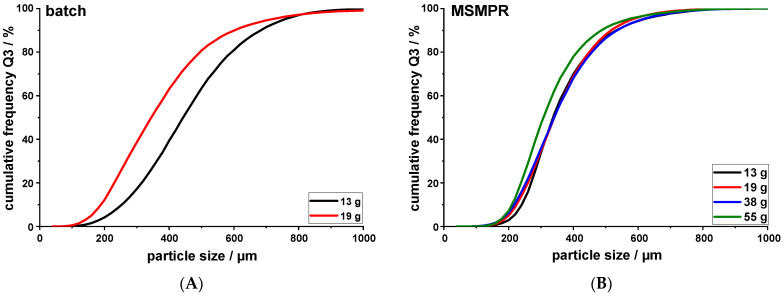
(**A**). Change in PSD of QESD MF agglomerates by increasing batch size using a batch crystallizer vs. (**B**). a MSMPR crystallizer (*n* = 1, entire batch).

The main aim of this work was to examine whether the developed MSMPR crystallizer could be used to increase the production capacity of a single run, while maintaining the PSD of the agglomerates. For this trial, the crystallizer was run at ${\dot{V}}_{\mathrm{API}}$ = 2.5 mL/min, ${\dot{V}}_{\mathrm{out}}$ = 23.80 mL/min, *V*_0_ = 600 g, and 350 rpm. As previously reported [8], increasing the volume of MF solution pumped into the crystallizer during batch production resulted in a shift to smaller particle sizes of the agglomerates (Figure 11A, EMD = 50). Using the MSMPR setup, this strong shift could not observed (Figure 11B). An EMD value of 19 was calculated between the 13 g and 55 g PSDs of the MSMPR, which was lower than the value calculated for the difference between the 13 g and 19 g produced in batch mode. The change in EMD was, however, higher than the value for the reproducibility trial (EMD = 4.2, Figure 6). The larger particle size of the agglomerates produced using the batch setup compared to the MSMPR setup (Figure 11) might be due to the agglomeration of particles in suspension, since a higher solid fraction was present during batch production, as material was not constantly removed from the system.

Replacing the mother liquor with fresh antisolvent in the MSMPR setup led to the formation of QESD agglomerates with similar particle sizes, while increasing the amount of API solution pumped into the system. However, looking at the values of the calculated average solvent fraction during the crystallization revealed that this cannot be the only reason for the observed phenomenon. While increasing the batch size from 13 g to 19 g, the calculated average solvent fraction increased from 1.38% to 1.92%. Increasing the batch size using the MSMPR setup led to an increase in the average solvent fraction (1.20% at 13 g, 1.60% at 19 g, 2.62% at 38 g, and 3.37% at 55 g), which even surpassed that of the batch trial; however, the EMD value remained lower. Nocent et al. [21] reported that no agglomerates could be formed at a SF > 1%, which was not the case for MF. This shows that this limit has to be set individually for each API, specifically for the respective solvents used.

The reduced shift in particle size when using the MSMPR setup might be explained by the position of the antisolvent and API feed line in the lid of the crystallizer (Figure 1). The addition of fresh antisolvent to the surface of the crystallizer was at a 90° offset to the API feed line against the stirring direction. This means that the MF solution droplets come into contact with fresh antisolvent when falling into the mother liquor. Furthermore, due to the addition of fresh antisolvent to the surface of the mother liquor, a decreased solvent fraction may be present in the upper layers of the mother liquor. As the crystallization of the particles could be observed within seconds, the layer of fresh antisolvent at the top of the crystallizer might counteract the effect of an increased average solvent fraction within the mother liquor. For further evaluation, a CFD simulation could be used to study the homogeneity of the mother liquor during mixing, and the position of the antisolvent feed varied to see if the effect is visible at different positions.

**Figure 12:**
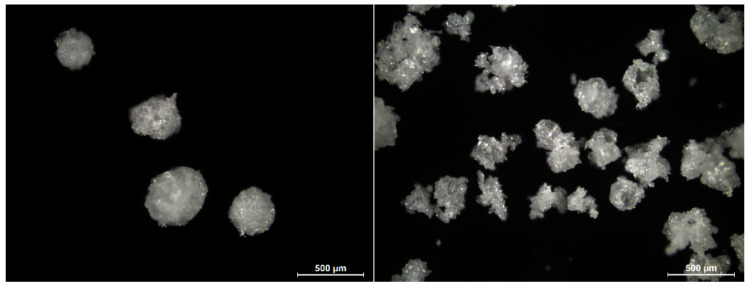
Microscopic images of QESD MF agglomerates after 50 mL ((**left**), average SF: 1.60%) and 150 mL ((**right**), average SF: 3.37%) of MF solution crystallized.

Looking at the morphology of the particles produced using the MSMPR setup revealed that those produced near the end of the run were less spherical in shape and had a rougher surface (Figure 12). The flowability of the batch remained good (Table 1), as the particles produced at the start of the batch can compensate for those produced later in the batch. The reduction in sphericity and surface smoothness of the particles may be due to the increased solvent fraction of the mother liquor. As the rate of diffusion decreases with increased solvent fraction, the degree of supersaturation at the interface of the transient emulsion droplets could be lower and the formation of a coherent, smooth crust through nucleation may be hindered. A reduced pump rate of the MF solution would have been beneficial; however, as mentioned previously, this was not feasible. Alternatively, it was calculated that an initial fill volume of 5300 mL would ensure a solvent fraction <1% at a ${\dot{V}}_{\mathrm{API}}$ = 2.5 mL/min and ${\dot{V}}_{\mathrm{out}}$ = 23.80 mL/min. However, this could also not be realized with the crystallizer at hand.

## 4. Conclusions

A typical lab-scale batch crystallizer could be converted into a simple MSMPR continuous crystallizer for quasi-emulsion solvent diffusion crystallizations of metformin hydrochloride. Mean residence times and the solvent fraction of the mother liquor could be calculated after verifying that the crystallizer behaved according to a one-compartment model with first-order kinetics. The findings of Tahara et al. [13] could be confirmed for a crystallizer running at approximately 100 times higher throughput. The particle size of the agglomerates could be changed by altering the mean residence time of the particles within the crystallizer. Batch sizes > 50 g were possible in a single run, without seeing a strong shift to smaller particle sizes, in contrast to the previously developed batch production. Further work should focus on implementing a second peristaltic pump, so that the antisolvent feed and product slurry removal can be controlled separately. This would allow for a constant fill level and, therefore, true steady-state conditions during crystallization. Furthermore, an optimization of the MF feed capillary should be performed, in order to allow for lower pump rates without blockage, and the effect of the position of the antisolvent feed tube within the crystallizer on the particle size of the QESD MF agglomerates should be studied.

## Figures and Tables

**Table 1:** Influence of batch size during MSMPR crystallization on the flowability of the QESD MF agglomerates (mean ± s, *n* = 3).

Batch Size/g	Hausner Ratio	Angle of Repose/°
13	1.17 ± 0.01	30.6° ± 0.6
19	1.15 ± 0.02	31.3° ± 0.6
38	1.12 ± 0.01	27.3° ± 0.6
55	1.22 ± 0.05	31.7° ± 0.6

## Data Availability

The data presented in this study are available in J. Hansen and P. Kleinebudde. Increasing the batch size of a QESD crystallization by using a MSMPR crystallizer. Pharmaceutics.
